# Transducin activates cGMP phosphodiesterase by trapping inhibitory γ subunit freed reversibly from the catalytic subunit in solution

**DOI:** 10.1038/s41598-019-43675-9

**Published:** 2019-05-10

**Authors:** Teizo Asano, Satoru Kawamura, Shuji Tachibanaki

**Affiliations:** 1 Graduate School of Frontier Biosciences, Osaka University, Yamada-oka 1-3, Suita Osaka, 565-0871 Japan; 2 Present Address: Medical Research Support Center, Graduate School of Medicine, Kyoto University, Yoshida-honmachi, Sakyo-ku, Kyoto 606-8501 Japan; 3 Department of Biological Sciences, Graduate School of Science, Osaka University, Yamada-oka 1-3, Suita Osaka, 565-0871 Japan

**Keywords:** Enzyme mechanisms, Retina

## Abstract

Activation of cGMP phosphodiesterase (PDE) by activated transducin α subunit (Tα*) is a necessary step to generate a light response in vertebrate photoreceptors. PDE in rods is a heterotetramer composed of two catalytic subunits, PDEα and PDEβ, and two inhibitory PDEγ subunits, each binding to PDEα or PDEβ. Activation of PDE is achieved by relief of the inhibitory constraint of PDEγ on the catalytic subunit. In this activation mechanism, it is widely believed that Tα* binds to PDEγ still bound to the catalytic subunit, and removes or displaces PDEγ from the catalytic subunit. However, recent structural analysis showed that the binding of Tα* to PDEγ still bound to PDEα or PDEβ seems to be difficult because the binding site of PDEγ to PDEα or PDEβ overlaps with the binding site to Tα*. In the present study, we propose a novel activation mechanism of PDE, the trapping mechanism, in which Tα* activates PDE by trapping PDEγ released reversibly and spontaneously from the catalytic subunit. This mechanism well explains PDE activation by Tα* in solution. Our further analysis with this mechanism suggests that more effective PDE activation in disk membranes is highly dependent on the membrane environment.

## Introduction

In the vertebrate photoreceptors, an enzymatic cascade, the phototransduction cascade, is responsible for generation of a light response^1,2^. Briefly, after absorption of light, light-activated visual pigment catalyzes the exchange of GDP for GTP on the α subunit of transducin (Tα) to produce a GTP-bound active form of transducin (Tα*). Tα* then activates cGMP phosphodiesterase (PDE). PDE is a heterotetrameric protein composed of two catalytic subunits of similar amino acid sequence (PDEα and PDEβ showing >70% sequence identity) and two inhibitory subunits (PDEγ), and therefore is in the form of PDEαγβγ. (We call this form of holo-PDE just PDE for simplicity.) Each catalytic subunit has an active site to hydrolyze cGMP to GMP. Tα* binds to inhibitory PDEγ, and relieves its constraint on the active site in the catalytic subunit. This activation of PDE causes hydrolysis of cGMP, leads to closure of cGMP-gated cation channels situated in the plasma membrane of the outer segment, and induces a hyperpolarization of the cell.

In the activation process of PDE by Tα*, it is widely believed that Tα* directly binds to PDEγ still bound to the catalytic subunit, and removes or displaces PDEγ from the active site of a catalytic subunit^3,4^. However, this mechanism seems to be difficult based on the recent structural studies on the PDEγ·PDEα complex and the PDEγ·Tα* complex: most of the amino acid residues in the C-terminal region of PDEγ, from Asp-63 to Ile-87, are in contact with Tα*^5^, and almost the same region, from Leu-60 to Ile-87 in PDEγ, is in contact with the catalytic site of PDEα or PDEβ^6^. These observations suggest that PDEγ utilizes the same region to bind to Tα* and to the catalytic site of PDEα or PDEβ, and that Tα* and the catalytic subunit cannot bind to this region simultaneously.

**Figure 1:**
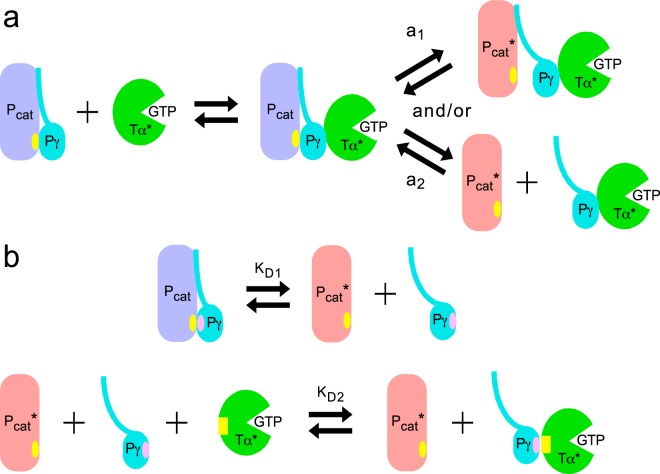
Possible PDE activation mechanisms. (**a**) Conventional mechanism. In the inactive state of PDE (purple), PDEγ (Pγ) binds to the PDE catalytic subunit (PDEα or β, indicated as Pcat) at the binding site on the catalytic subunit (yellow oval). Activated Tα (Tα*) binds to PDEγ to displace (a_1_) and/or remove PDEγ (a_2_) from the catalytic subunit to activate PDE (pale red). (**b**) Trapping mechanism. PDEγ is bound to the catalytic site of PDE (yellow oval) with the binding site in PDEγ (pink oval), but PDEγ is freed reversibly from the catalytic subunit according to the dissociation constant, K_D1_ (upper). This freed PDEγ is trapped by Tα* with the dissociation constant, K_D2_, at the binding site of PDEγ (pink oval) to Tα* (yellow rectangular) to inhibit re-binding of PDEγ to the catalytic subunit (lower).

These considerations led us to examine a novel mechanism of PDE activation in vertebrate photoreceptors (Fig. 1). In the conventional activation mechanism (Fig. 1a), Tα* binds to PDEγ (Pγ) still bound to the catalytic subunit (Pcat), and displaces (a_1_ in Fig. 1a) or removes PDEγ (a_2_) from the catalytic subunit to activate PDE. (We assume that PDEα and PDEβ behave indistinguishably, and call either of them PDEcat in the following.) In the novel mechanism (Fig. 1b), PDEγ is freed from PDEcat reversibly according to the dissociation constant of K_D1_ of the complex of PDEγ·PDEcat. Tα* then traps freed PDEγ with the dissociation constant of K_D2_ of the complex of PDEγ·Tα* to activated PDE (trapping mechanism). In the present study, therefore, we determined K_D1_ and K_D2_, and examined whether one can explain PDE activation at various concentrations of Tα* using an equation formulated for the trapping mechanism. The result reasonably explained PDE activation caused by addition of various concentrations of Tα* in solution.

## Results

### Much more effective binding of Tα-S* to free PDEγ than to PDEγ still bound to PDEcat

**Figure 2:**
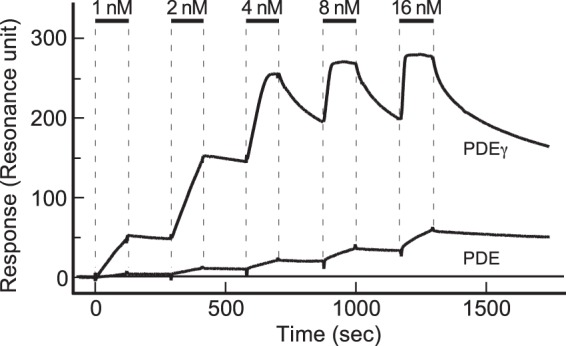
Comparison of the bindings of PDEγ and PDE to immobilized Tα-S* with the SPR method. Recombinant PDEγ or purified PDE was injected at the concentrations indicated. Injections were made as indicated (horizontal bars), and bound proteins were washed out after each injection. Immobilization level of Tα-S* was ~2300 resonance unit (RU).

To make sure that Tα* binds much more effectively to free PDEγ than to PDEγ still bound to PDEcat, we measured the binding of recombinant free PDEγ and that of purified PDE to Tα* with the Surface Plasmon Resonance (SPR) method. For this, we used the guanosine 5′-O-(γ-thio) triphosphate (GTPγS)-bound form of Tα (Tα-S*) as Tα*, and immobilized it on the surface of an SPR sensor chip as the common binding target of free PDEγ and PDEγ still bound to PDEcat. Figure 2 shows a series of association-dissociation time courses of recombinant PDEγ and that of PDE (i.e., PDEγ·PDEcat complex), both at 1–16 nM (horizontal bars). As seen, the binding signal is much larger with free PDEγ than with PDE at all concentrations examined. Note that these measurements were made on the same sensor chip, so that we can compare the binding signals directly at each concentration of PDEγ and PDE. The other point is that the SPR signal is proportional to the mass bound to the immobilized protein. The molecular mass of PDEγ is 9.5 kDa and that of PDE is 216.4 kDa. When the same number of PDE molecules binds to the sensor chip as that of PDEγ, the signal of PDE should be 23 times (216.4/9.5) larger than that of PDEγ. The result in Fig. 2, therefore, showed that free PDEγ binds to Tα-S* much more effectively than PDE, which is inconsistent with the conventional mechanism a_1_ in Fig. 1a.

We measured the binding signals using a running buffer that did not contain cGMP throughout our study. It is well known that PDEcat has one or two non-catalytic cGMP binding sites^7^. When these non-catalytic sites are empty, which is most likely with our purified PDE used, Tα* physically removes PDEγ from PDEcat upon activation^8^. Thus, on injection of PDE, we could expect that PDEcat is removed from PDEγ that has been associated with immobilized Tα-S* on the sensor chip. Then, we could expect that the binding signal of PDE is almost the same as that of recombinant PDEγ of the same concentration, which is not the case in Fig. 2. Therefore, the result in Fig. 2 is also inconsistent with the conventional mechanism a_2_ in Fig. 1a.

The binding signal of PDE in Fig. 2 suggests two possibilities: (i) Tα-S* binds to PDEγ still bound to PDEcat much more weakly than free PDEγ or (ii) Tα-S* traps limited amount of PDEγ freed reversibly and spontaneously from PDE. Based on the possibility (i), in Fig. 2, we estimated the bound recombinant PDEγ/PDE molar ratio at the time point of 1500 sec, and it was 83/1. In other words, the affinity of Tα-S* to free PDEγ is higher than that of PDEγ in PDE by almost two orders of magnitude. For this reason, we thought that the above possibility (i) is not plausible, and decided to examine the 2nd possibility, the trapping mechanism.

### Formulation of the trapping mechanism

According to the reaction scheme shown in Fig. 1b, we formulated Eq. (1), same as equation (s10) in SI Methods, which expresses the PDE activity as a function of Tα* concentration (Tα-S* concentration in this study):

1 $$\begin{array}{c}({{\rm{K}}}_{{\rm{D}}1}-{{\rm{K}}}_{{\rm{D}}2}){[{\rm{P}}{\rm{D}}{\rm{E}}{\rm{c}}{\rm{a}}{\rm{t}}]}^{3}+({{{\rm{K}}}_{{\rm{D}}1}}^{2}-{{\rm{K}}}_{{\rm{D}}1}\,{{\rm{K}}}_{{\rm{D}}2}-2\,{{\rm{K}}}_{{\rm{D}}1}\,[{\rm{P}}{\rm{D}}{\rm{E}}{\rm{c}}{\rm{a}}{\rm{t}}]{\rm{t}}{\rm{o}}{\rm{t}}{\rm{a}}{\rm{l}}\\ +{{\rm{K}}}_{{\rm{D}}1}[{\rm{P}}{\rm{D}}{\rm{E}}\gamma ]{\rm{t}}{\rm{o}}{\rm{t}}{\rm{a}}{\rm{l}}-{{\rm{K}}}_{{\rm{D}}1}[{{\rm{T}}\alpha }^{\ast }]{\rm{t}}{\rm{o}}{\rm{t}}{\rm{a}}{\rm{l}}\\ +{{\rm{K}}}_{{\rm{D}}2}[{\rm{P}}{\rm{D}}{\rm{E}}{\rm{c}}{\rm{a}}{\rm{t}}]{\rm{t}}{\rm{o}}{\rm{t}}{\rm{a}}{\rm{l}}-{{\rm{K}}}_{{\rm{D}}2}\,[{\rm{P}}{\rm{D}}{\rm{E}}\gamma ]{\rm{t}}{\rm{o}}{\rm{t}}{\rm{a}}{\rm{l}}){[{\rm{P}}{\rm{D}}{\rm{E}}{\rm{c}}{\rm{a}}{\rm{t}}]}^{2}\\ -{{\rm{K}}}_{{\rm{D}}1}\,[{\rm{P}}{\rm{D}}{\rm{E}}{\rm{c}}{\rm{a}}{\rm{t}}]{\rm{t}}{\rm{o}}{\rm{t}}{\rm{a}}{\rm{l}}(2\,{{\rm{K}}}_{{\rm{D}}1}-{{\rm{K}}}_{{\rm{D}}2}-[{\rm{P}}{\rm{D}}{\rm{E}}{\rm{c}}{\rm{a}}{\rm{t}}]{\rm{t}}{\rm{o}}{\rm{t}}{\rm{a}}{\rm{l}}\\ +[{\rm{P}}{\rm{D}}{\rm{E}}\gamma ]{\rm{t}}{\rm{o}}{\rm{t}}{\rm{a}}{\rm{l}}-[{{\rm{T}}\alpha }^{\ast }]{\rm{t}}{\rm{o}}{\rm{t}}{\rm{a}}{\rm{l}})[{\rm{P}}{\rm{D}}{\rm{E}}{\rm{c}}{\rm{a}}{\rm{t}}]+{({{\rm{K}}}_{{\rm{D}}1}[{\rm{P}}{\rm{D}}{\rm{E}}{\rm{c}}{\rm{a}}{\rm{t}}]{\rm{t}}{\rm{o}}{\rm{t}}{\rm{a}}{\rm{l}})}^{2}=0\end{array}$$

where K_D1_ and K_D2_ are the dissociation constants of the PDEγ·PDEcat complex and of the PDEγ·Tα* complex, respectively (Fig. 1b); [PDEcat]total, [PDEγ]total and [Tα*]total are the total concentrations of PDEcat (namely, the concentration of PDEα plus PDEβ), PDEγ and Tα*, respectively. The concentrations of [PDEcat]total, [PDEγ]total and [Tα*]total are known values in our measurement of the PDE activity, and K_D1_ and K_D2_ are the only unknown parameters in the above equation. In the followings, we tried to determine these values experimentally, and examined whether we can explain PDE activities elicited by addition of Tα-S* of known concentration. Because the effectiveness of Tα-S* on PDE activation was different in the measurement using purified PDE and that using PDE in rod outer segment (ROS) membranes, we examined both cases in the followings.

### Determination of K_D1_ of the complex of PDEγ and PDEcat

**Figure 3:**
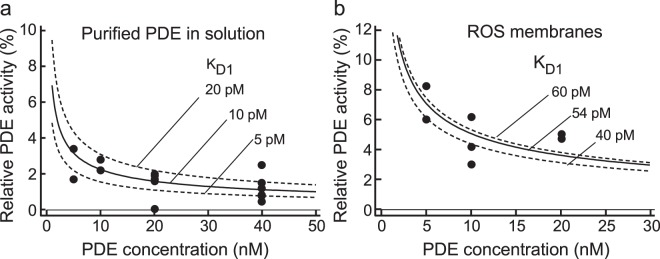
Determination of K_D1_ of the PDEγ·PDEcat complex. PDE activity was measured using purified PDE (**a**) and ROS membranes (**b**) at the concentrations of PDE shown in the horizontal axis. Each data point shows the result of a single activity measurement. The activity is shown as the relative activity (%) against the full PDE activity that was determined after treatment with trypsin (trypsin-treated). (**a**) The concentration of purified PDE was calibrated with SDS-PAGE. The relation between relative PDE activity and the concentration of PDE was fitted with equation (s5) to determine K_D1_ of the PDEγ·PDEcat complex. The best-fitted K_D1_ in solutions of purified PDE was 10 pM (solid curve), and the expected curve for K_D1_ of 5 pM and that of 20 pM are also shown (broken curves). (**b**) Similar as in (**a**), but PDE content in a ROS membrane suspension was estimated by assuming that the molar ratio of PDE to rhodopsin is 1/270 in ROS membranes^14^. The best-fitted K_D1_ was 54 pM (solid curve). Expected curve for K_D1_ of 40 pM and that of 60 pM are also shown (broken curves).

To determine K_D1_ of the PDEγ·PDEcat complex, first we measured the PDE activity using purified PDE at diluted low concentrations (Fig. 3a, filled circles). As dilution increases, the concentration of freed PDEγ, and therefore, relative PDE activity increases depending on K_D1_. The relation between the concentration of PDE and the measured relative PDE activity was fitted with an equation formulated for a simple binding-dissociation reaction of PDEγ and PDEcat (equation (s5) in SI Methods) to determine K_D1_. The best-fitted K_D1_ of the PDEγ·PDEcat complex in purified PDE was 10 pM. However, the data points scattered slightly in Fig. 3 so that we could only determine the range of K_D1_: it was approximately 5–20 pM (broken curves for K_D1_ of 5 and 20 pM) and close to the reported value of <10 pM obtained with purified bovine PDE previously^4^. It should be mentioned here that freed PDEγ is completely freed from PDEcat. If freed PDEγ is removed from the active site but is still attached to PDEcat, dilution will not induce the increase in the relative PDE activity. It is because dilution does not affect re-binding of PDEγ to PDEcat in this case.

Similar dilution study was made using ROS membranes (filled circles in Fig. 3b). The best-fitted K_D1_ of the PDEγ·PDEcat complex in ROS membranes was 54 pM, and the range was 40–60 pM (broken curves for K_D1_ of 40 and 60 pM). Postulated elution of freed PDEγ in the dark in ROS membrane suspensions was examined with washing membranes, and the resultant increase in PDE activity was observed with repetitive washes (Fig. S1).

### Determination of K_D2_ of the complex of PDEγ and Tα-S*

**Figure 4:**
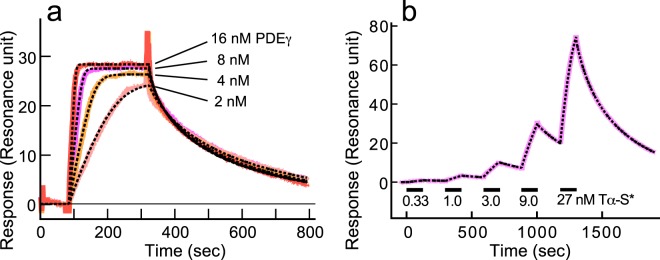
Determination of K_D2_ of the PDEγ·Tα-S* complex. (**a**) SPR measurements of the binding of PDEγ to immobilized Tα-S*. Recombinant PDEγ was injected at various concentrations indicated, and perfused until the binding signal was almost saturated. The bound proteins were washed out almost completely after each of the injections. Immobilization level of Tα-S* was ~400 RU. The binding signals (solid traces) were fitted globally using a Heterogeneous Ligand with MTL program to calculate K_D2_, and it was 0.73 ± 0.13 nM (mean ± SE, n = 3). Flow rate was 10 μl/min. (**b**) SPR measurements of the binding of Tα-S* to immobilized PDEγ. Tα*-S was injected at indicated concentrations and perfused for 125 sec (horizontal bars) for the binding and then washed out for 175 sec each time. The binding signal (pink solid trace) was globally fitted using a 1:1 binding with MTL program (black broken trace) to calculate K_D2_. The best-fitted K_D2_ was 5.6 ± 1.3 nM (mean ± SE, n = 5). Immobilization level of PDEγ was ~100 RU. Flow rate was 30 μl/min.

To measure K_D2_ of the PDEγ·Tα-S* complex, we measured it in two configurations (Fig. 4) using the SPR method. One configuration was similar to that shown in Fig. 2: Tα-S* was immobilized. In Fig. 4a, 2–16 nM PDEγ was perfused until the signal reached to a steady level and bound PDEγ was washed out almost completely at each PDEγ concentration. All of the measured time courses were then globally fitted with a program provided by the manufacturer (black broken traces in Fig. 4a, see Methods) to determine K_D2_ of the PDEγ·Tα-S* complex. In a total of three different measurements using two different sensor chips, we obtained K_D2_ of 0.73 ± 0.13 nM (mean ± SE, n = 3) for the PDEγ·Tα-S* complex.

The value of K_D2_ was determined in the reversed configuration: PDEγ was immobilized and Tα-S* was perfused (Fig. 4b). In this case, Tα-S* of increasing concentration was perfused in a less-time consuming way: Tα-S* was added before bound Tα-S* was washed out completely (pink trace). Measured time course was fitted with the other program provided by the manufacturer (black broken trace in Fig. 4b, see Methods). From the fitting results, K_D2_ was estimated to be 5.6 ± 1.3 nM (mean ± SE, n = 5). (According to the manufacturer’s protocol, the same dissociation constant can be obtained no matter whether bound protein is completely washed out as in Fig. 4a or not as in Fig. 4b.)

Obtained values of K_D2_ in two configurations (Tα-S* immobilized or PDEγ immobilized) were ~8 times different (0.73 nM/5.6 nM = 1/7.7). Although immobilizations of Tα-S* and PDEγ were designed not to affect the binding site seriously (see Methods), immobilization seemed to affect K_D2_ slightly. We, therefore, concluded that K_D2_ is 0.73–5.6 nM, which is consistent with the values of 0.1–33 nM reported previously utilizing various methods for the measurement^4,8–10^.

### Validation of the trapping mechanism for PDE activation of purified PDE in solution

In Figs 3 and 4, we determined the ranges of K_D1_ of the PDEγ·PDEcat complex for purified PDE (Fig. 3a) and PDE in ROS membranes (Fig. 3b), and the range of K_D2_ of the PDEγ·Tα-S* complex (Fig. 4) using the SPR method. To validate the trapping mechanism, we then examined whether this mechanism can explain the activation of PDE by Tα-S* of known concentrations with use of Eq. (1) formulated for this mechanism.

**Figure 5:**
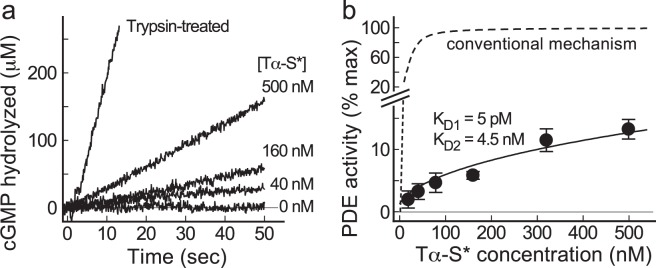
Activation of purified PDE by purified Tα-S* in a solution. (**a**) Sample traces of PDE activity measurement by the pH assay method in a solution containing ~15 nM PDE and various concentration of Tα-S* indicated. Full PDE activity was determined after treatment with trypsin (trypsin-treated). (**b**) PDE activation as a function of concentration of Tα-S* added. Vertical axis shows the % of activation. Each data point is a mean ± SE (n = 6 except for the point at 10 nM Tα-S*, where n = 3). The data points were fitted with Eq. (1) formulated under the conditions that PDE is activated through the trapping mechanism with K_D1_ = 5 pM and K_D2_ = 4.5 nM (see text). Broken line shows the theoretical curve with the conventional activation mechanism, where K_D_ = 4.5 nM (see text).

Figure 5a shows the measurement of activation of purified PDE by purified Tα-S* in solution at indicated concentrations with the pH assay method^11,12^. The pH decrease accompanied by hydrolysis of cGMP was calibrated, and the PDE activity was determined from the slope. Full PDE activity was determined after treatment with trypsin (trypsin-treated). PDE activity at a given Tα-S* concentration is expressed as the % of the full PDE activity, and the summarized result is shown in Fig. 5b (filled circles and bars showing mean ± SE). Then, the relation between the relative PDE activity and the Tα-S* concentration was fitted with Eq. (1). As shown above, we determined the range of K_D1_ in solution (5–20 pM, Fig. 3a) and that of K_D2_ (0.73–5.6 nM, Fig. 4), and for this reason, we tried to examine whether we can explain PDE activation by Tα-S* in Fig. 5a with these dissociation constants in those ranges. First, we used K_D1_ of 10 pM, but could not obtain a best-fitted value of K_D2_ within the range of K_D2_ we determined in Fig. 4. For this, we set K_D1_ at 5 pM, for example, and then determined K_D2_ that provides the best fit to the PDE activation curve. The value of K_D1_ was increased by 1 pM step and the best-fitted K_D2_ was determined each time. We then found that at each K_D1_ value from 2 pM to 6 pM, we can find a K_D2_ value that gives a reasonable fit to the PDE activation curve in Fig. 5b. Interestingly, each pair of K_D1_ and K_D2_ we determined showed similar goodness of fit (χ^2^, Table 1), and we show the result of K_D1_ = 5 pM and K_D2_ = 4.5 nM in Fig. 5b (solid curve). We tried to estimate the activation curve of PDE by Tα-S* with the conventional binding mechanism shown in Fig. 1a under the condition of K_D_ = 4.5 nM. The expected curve deviated greatly from the measured result (broken curve in Fig. 5b). From this result, we concluded that Tα-S* activates PDE by trapping PDEγ freed reversibly from PDE and by inhibiting its re-binding to PDEcat. An alternative possibility is that Tα-S* binds to PDE (PDEαβ or PDEγ still bound to PDEαβ). However, this possibility can be excluded because the binding signal of PDE to Tα-S* is low (Fig. 2). PDE activation with use of purified PDE in solution was measured only at 15 nM PDE. It was because at higher concentrations of purified PDE, the measurement was not possible because of protein aggregation.

**Table 1:** Fitted results of K_D2_ for purified PDE activation by Tα-S* in solution.

K_D1_ (constant, pM)	K_D2_ (fitted, nM)	χ^2^
2.0	1.8	0.000546
3.0	2.7	0.000576
4.0	3.6	0.000611
5.0	4.5	0.000648
6.0	5.5	0.000688
7.0	6.4	0.000729
8.0	7.3	0.000772

### Validation of the trapping mechanism for activation of PDE in ROS membrane suspension

**Figure 6:**
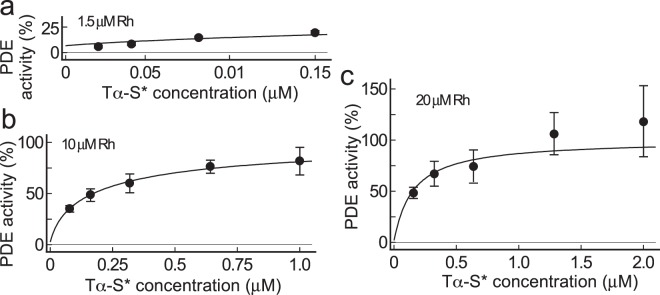
Activation of PDE with Tα-S* in ROS membrane. Percentage of PDE activation is shown as a function of Tα-S* concentration in suspensions of (**a**) 1.5, (**b**) 10 and (**c**) 20 μM rhodopsin membranes. Each data point is a mean ± SE (n = 3–6). The data points were fitted by Eq. (1) with fixed K_D1_ (55 pM, see text) and K_D2_ of 23.6 nM (**a**), 0.201 nM (**b**) and 0.0565 nM (**c**).

As shown above, it is highly possible that purified PDE is activated by the trapping mechanism in solution. Then we examined whether this mechanism is applied to PDE activation in ROS membranes. In the measurement of PDE activity in an illuminated ROS membrane suspension, we added GTPγS at a concentration lower than that of Tα, of which concentration was estimated on the assumption that the molar ratio of Tα to rhodopsin^13^ is 1/10. In this way, we limited the amount of Tα-S* by the amount of added GTPγS^12^. In our previous study^12^, PDE activation by addition of GTPγS is dependent on the ROS membrane concentration: the lower the concentration, the lower the maximum PDE activation. For this reason, ROS membranes containing rhodopsin of 1.5, 10 and 20 μM (abbreviated as 20 μM rhodopsin membranes, for example) were used to measure the PDE activation at various concentrations of Tα-S*. The activity was measured similarly as in Fig. 5a, and the results are shown in Fig. 6a–c (circles and bars showing mean ± SE). Note that the horizontal axis is different in each panel, which is because the maximum Tα-S* concentration should be equal to the concentration of Tα at different membrane concentrations (0.15 μM Tα in 1.5 μM rhodopsin membranes, for example). As reported previously^12^, in 20 μM rhodopsin membranes, we obtained almost a full PDE activity that is observed in trypsin-treated ROS membranes (Fig. 6c). Then, we fitted the results in Fig. 6 with Eq. (1) to estimate K_D1_ and K_D2_ in ROS membranes at each membrane concentration. As shown in Fig. 3b, we found that the range of K_D1_ in ROS membranes is in the range of 40–60 pM. We therefore arbitrary set K_D1_ at 40–60 pM with 5 pM step, and determined K_D2_ each time. The results are summarized in Table 2. Each pair of K_D1_ and K_D2_ in Table 2 gave reasonable fit to the PDE activation curve in ROS membranes without significant differences at each ROS membrane concentration (see χ^2^ for each membrane concentration in Table 2). Because we obtained the value of 54 pM as K_D1_ in ROS membranes (Fig. 3b), fitting result with K_D1_ of 55 pM is shown at each membrane concentration (Fig. 6). We assume the presence of freed PDEγ in the dark or in the absence of Tα-S*, and our fitting showed that its population is low and 1.9% in 20 μM rhodopsin membranes (Fig. 6c).

**Table 2:** Fitted results of K_D2_ for PDE activation by Tα-S* in ROS membrane suspension.

[Rhodopsin] (μM)	K_D1_ (constant, pM)	K_D2_ (fitted, nM)	χ^2^
1.5 μM	40.0	15.9	0.00425
	45.0	18.3	0.00466
	50.0	20. 9	0.00508
	55.0	23.6	0.00551
	60.0	26.4	0.00595
10 μM	40.0	0.147	0.000786
	45.0	0.164	0.000844
	50.0	0.182	0.000845
	55.0	0.201	0.000845
	60.0	0.219	0.000846
20 μM	40.0	0.0410	0.0332
	45.0	0.0462	0.0332
	50.0	0.0513	0.0332
	55.0	0.0565	0.0332
	60.0	0.0617	0.0332

In Table 2, best-fitted K_D2_ values varied significantly depending on the ROS membrane concentration, and they decrease as membrane concentration increases: at a constant K_D1_ value of 55 pM, the best-fitted K_D2_ values are 23.6, 0.201 and 0.0565 nM in 1.5, 10 and 20 μM rhodopsin membranes, respectively. Apparent ROS membrane concentration-dependent changes in K_D2_ suggest the loss of intrinsic Tα-S* from membranes (see Discussion). Although K_D2_ values in 1.5 and 20 μM rhodopsin membranes (23 and 0.0565 nM, respectively) were not in the K_D2_ range we observed in Fig. 4 in solution (SPR study, 0.73–5.6 nM), our analysis seemed to explain PDE activation by Tα-S* in ROS membranes with the trapping mechanism as well (but, see Discussion). We estimated the PDEcat activation at pseudo-physiological concentrations of Tα and PDEcat with the trapping mechanism. For this, we assumed that the rhodopsin concentration is 3 mM and that the transducin^13^ and PDE^14^ contents are 1/10 and 1/270, respectively, of that of rhodopsin. As a result, at 0.3 mM Tα* (full Tα activation) and 22.2 µM PDEcat, and with K_D_ values in membranes (K_D1_ = 55 pM and K_D2_ = 56.5 pM), we obtained the result that 93% of PDEcat is activated in membranes. This high activation of PDE at these Tα* and PDEcat concentrations was not obtained with use of K_D_ values we obtained in solution (K_D1_ = 5 pM and K_D2_ = 4.5 nM), and the PDEcat activation in this case was only ~11%. The result showed that PDE activation is different between in solution and in membranes, and that one cannot apply the K_D_ values obtained in solution directly to the study on PDE activation in membranes.

## Discussion

In the activation mechanism of photoreceptor PDE, it has been generally believed that Tα* binds to PDE, and removes or displaces PDEγ from the catalytic site of PDEcat (conventional mechanism, Fig. 1a). However, our SPR analysis showed that Tα* (actually Tα-S*) binds much more effectively to PDEγ than to PDE (Fig. 2), which is not consistent with the mechanism a_1_ in Fig. 1a. The result in Fig. 2 also revealed that Tα* hardly releases PDEγ from PDE, which shows that the mechanism a_2_ shown in Fig. 1a is unlikely (Fig. 2). Furthermore, with the conventional activation mechanism, Tα*-dependent PDE activation could not be explained quantitatively in solution (Fig. 5b). Instead, PDE activation in solution is reasonably explained by the trapping mechanism (Fig. 5 and Table 1) using experimentally estimated dissociation constants of the PDEγ·PDEcat complex (K_D1_, Fig. 3a) and the PDEγ·Tα* complex (K_D2_, Fig. 4).

In the fitting of PDE activation in ROS membrane suspension, best-fitted K_D2_ decreased as the membrane concentration increased: at a constant value of K_D1_ of 55 pM, K_D2_ was 23.6 nM in 1.5 μM rhodopsin membranes, and it decreased significantly to 0.0565 nM in 20 μM rhodopsin membranes (Table 2). Apparently, K_D2_ that can be determined in a ROS membrane suspension is dependent on the membrane concentration. The reason for this is not known. However, we previously found that ~65% Tα-S* is eluted from 0.75 μM rhodopsin membranes, but ~50% from 15 μM rhodopsin membranes in carp^12^. This 15% excess in the amount of Tα-S* remaining in 15 μM rhodopsin membranes seems to be sufficient to activate all of PDEcat molecules^12^, which could be deduced by the Tα/rhodopsin molar ratio^13^ of 1/10 and the PDE/rhodopsin (i.e., 1/2PDEcat/rhodopsin) molar ratio^14^ of 1/270: the molar ratio of 15% of Tα-S* to PDEcat in ROS membranes is ~2:1. It is possible that there could be two types of Tα*. One type binds to membranes tightly and the other loosely, and the latter re-binds to the membrane effectively when the membrane concentration is high. We speculate that the loosely-bound Tα* becomes soluble rather easily at low membrane concentrations to increase K_D2_ and to reduce the maximum PDE activation by reducing the effective Tα* concentration. At high membrane concentrations, this type of Tα* re-binds effectively to the membranes to contribute significantly to activate PDEcat and to lower K_D2_. In fact, Tα has been known to be differentially lipidated with 65% of unsaturated and 30% of saturated C12 or C14 fatty acids^15^.

The trapping mechanism explains PDE activation in solution with K_D1_ and K_D2_, both determined experimentally (Fig. 5). It also explains the activation of PDE in ROS membranes with K_D1_ obtained experimentally in membranes and K_D2_ estimated by a fitting (Fig. 6). According to the measured K_D_ values in membranes, the affinity of PDEγ to PDEcat (K_D1_ = 55 pM, Fig. 3b) is almost the same as that to Tα* (K_D2_ = 56.5 pM, Fig. 6c), which is consistent with a very effective activation of PDE by Tα-S* in membranes. In contrast, K_D1_ is lower (5 pM) and K_D2_ is higher (4.2 nM) in solution than those in membranes, which would be the reason why PDE activation by Tα-S* is not so efficient in solution (Fig. 5b). One possible reason for these differences in K_D_ values in membranes and in solution would be the difference in protein conformation in membranes and in solution.

We further examined whether we can expect sufficiently large PDE activation at a level of a single photon response with the trapping mechanism. Using Eq. (1) and expected Tα* concentration necessary for generation of the response together with K_D1_ and K_D2_ values obtained in membranes, we found that 6.32% of PDEcat, is active in a single surface of a disk membrane. In contrast, in case no Tα* is present, i. e., in the dark, 0.16% of PDEcat is active (for details, see SI methods). This result seems to indicate that the trapping mechanism can be applied also to PDE activation in membranes.

However, the values of K_D2_ in ROS membranes were not determined experimentally and further we are not sure how we can apply K_D1_ and K_D2_ values to the activation of PDE in membranes. Tα* and PDE in a disk membrane are undoubtedly situated at certain orientations on the disk membrane, which probably increases the chance of encounter of Tα* to PDEγ. Additionally, membrane proteins (for example, PDEcat) are localized only in disk membranes while soluble proteins (Tα-S* and freed PDEγ) are in an aqueous phase in our measurement in ROS membrane suspensions. It is not certain whether we can appropriately apply Eq. (1) to PDE activation in these cases. Further complication seems to be present when we want to extend our analysis to PDE activation in intact ROS. Freed PDEγ is supposedly present in the inter-diskal space of which volume is of the order of <1 fL, where re-binding of freed PDEγ to PDEcat would be much more effective compared with the re-binding in a test tube. For these reasons, further studies seem to be necessary to find how we can apply the trapping mechanism to PDE activation in intact ROS from kinetical and mechanistic view point. Nonetheless, because PDEγ binds to PDEcat or Tα* using the same region, Tα* should bind to PDEγ after the dissociation or displacement of PDEγ from PDEcat even when the dissociation or displacement is induced after multistep interaction between Tα* and PDEγ as suggested^16,17^.

## Methods

### Preparation of rod outer segment (ROS) membranes from frog

All experiments with frogs (*Rana catesbeiana*) in this study were performed in accordance with the institutional guidelines and all experimental protocols were approved by Osaka University Graduate School of Frontier Biosciences (approval number FBS-15-003). ROS membranes were prepared as described previously using a stepwise sucrose density gradient^18^. Obtained ROS membranes were frozen in liquid nitrogen and stored at −80 °C until use. To calibrate the concentration of ROS membranes, the amount of rhodopsin in an aliquot of the membranes was quantified spectrophotometrically with assuming that the molar absorption coefficient of frog rhodopsin is 40,000 M^−1^ cm^−1^ at 500 nm. All of these manipulations were carried out in complete darkness with the aid of an infrared image converter (NVR 2015; NEC, Tokyo, Japan).

### Extraction and Purification of PDE and Tα-S* from ROS membranes

Crude PDE and crude Tα-S* were extracted basically as described previously^12,19^. Crude PDE was then loaded on a Mono Q PC 1.6/5 column (ÄKTAmicro system, GE Healthcare), and a 0–1 M NaCl gradient in an elution buffer (10 mM HEPES-NaOH, 2 mM MgCl_2_, 1 mM DTT, pH7.5) containing 0.005% (v/v) Tween 20 was applied. Eluted fractions at 0.47–1 M NaCl containing purified PDE were concentrated using a Spin-X UF column (Mr 30,000 cutoff, Corning). Then, the buffer containing purified PDE was changed to a potassium gluconate buffer (K-gluc buffer; 115 mM potassium gluconate, 10 mM HEPES, 2.5 mM KCl, 2 mM MgCl_2_, 0.2 mM EGTA, 0.1 mM CaCl_2_, and 1 mM dithiothreitol (DTT), pH 7.5) containing 0.005% (v/v) Tween 20 using a Superdex 200 PC 10/300 GL column (ÄKTAmicro system, GE Healthcare). The resultant purified PDE solution was concentrated using a Spin-X UF column, and stored at −80 °C until use. An aliquot of purified PDE was subjected to SDS-PAGE and the gels were stained with Oriole Fluorescent Gel Stain Kit (Bio-Rad) to assess the purity of PDE and also to quantify its amount using bovine serum albumin as a molar standard. Purity of PDE was almost 100% and the molar ratio of 2PDEγ/PDEαβ was 1.01 ± 0.01 (mean ± SE, n = 3).

Tα-S* was purified from crude Tα-S* according to the method reported previously^19^. Briefly, a Blue Sepharose 6 Fast Flow column (GE Healthcare) and a DEAE Sepharose Fast Flow column (GE Healthcare) were connected in tandem in this order for the purification. Then, a solution of crude Tα-S* supplemented with 2 mM MgCl_2_ was loaded on the column equilibrated with the elution buffer. The column was washed with the elution buffer sufficiently to remove unbound proteins, and then the Blue Sepharose column and the DEAE Sepharose column were separated: frog Tβγ bound to the Blue Sepharose column, and most of Tα-S* passed through this column and bound to the DEAE Sepharose column. Thus, Tα-S* bound to the DEAE Sepharose column was eluted using a 0–1 M NaCl gradient in the elution buffer. Tα-S* was then concentrated using a Spin-X UF column (Mr 10,000 cutoff, Corning). The buffer was changed to K-gluc buffer containing 0.005% (v/v) Tween 20 using a Superdex 75 PC 3.2/30 column (ÄKTAmicro system, GE Healthcare). Purified Tα-S* was stored at −80 °C until use. Purity and the concentration of Tα-S* were assessed with SDS-PAGE, and the purity was almost 100%. All of the manipulations for extraction and purification were performed at 4 °C.

### Expression and purification of recombinant PDEγ

DNA sequence of frog PDEγ (GenBank Accession Number AB578858.1) was inserted into NdeI/BamHI sites of expression vector, pET-3a (Novagen). PDEγ was expressed in E. coli BL21(DE3) pLysS strain (Novagen) after induction with IPTG for 3 hr at 30 °C. Purification of expressed PDEγ was carried out based on the method described previously^20,21^. Purified recombinant PDEγ was lyophilized, dissolved in K-gluc buffer and stored at −80 °C until use.

### Measurement of binding of PDEγ or PDE to immobilized Tα-S* and that of Tα-S* to immobilized PDEγ

Proteins were immobilized on the SPR sensor tip according to the protocol described by Biacore. To immobilize Tα-S* on the sensor chip, Tα-S* was first biotinylated at its thiol groups. For this purpose, 3.3 μl of 2 mM EZ-Link Maleimide-PEG2-Biotin (Thermo Fisher Scientific) was added to 100 μl of 13 μM of purified Tα-S* in K-gluc buffer without DTT, and the mixture was incubated for 1 hr on ice. After the incubation, 0.35 μl of 1 M DTT was added to reduce and deactivate the non-reacted maleimide group of Maleimide-PEG2-Biotin. Then, the buffer was changed to K-gluc buffer to remove the deactivated Maleimide-PEG2-Biotin using a Zeba Spin Desalting Column (Thermo Fisher Scientific). Purified biotinylated Tα-S* was immobilized on a streptavidin (SA) sensor chip (GE Healthcare) through the streptavidin-biotin interaction. There are 8 thiol groups in bovine Tα (NCB Accession # NM_181022.2). However, it is known that only one group is chemically modified by N-ethylmaleimide in the GTP-bound form of Tα and that this modification does not affect PDE activation^22^, and therefore the binding to PDEγ. Frog Tα also contains 8 thiol groups (NM_001090561.1), and our Tα-S* showed almost a single component in the binding to PDEγ (see below).

Recombinant PDEγ was immobilized at its lysine amino groups on a carboxymethylated dextran (CM5) sensor chip (GE Healthcare) using 1-ethyl-3-(3-dimethylaminopropyl)-carbodiimide (EDC) and N-hydroxysuccinimide (NHS). Unreacted NHS-ester was blocked with ethanolamine after the immobilization. There are 8 lysine residues in PDEγ, and all of them are at the region outside of the major binding site of PDEγ to Tα-S* and to PDEcat, but four of them are near the possible binding site of PDEγ to Tα*and PDEcat. However, these four did not seem to affect the binding (see below).

The binding of a protein to the immobilized protein was measured using Biacore X100 (GE Healthcare) at 25 °C. The buffer used was K-gluc buffer containing 0.005% (v/v) Tween 20. Binding signals were stored and processed in Biacore X100. We used two ways to record the binding, one with binding and dissociation both terminated before their completion (Figs 2 and 4b) and the other after their completion (Fig. 4a).

When necessary, the binding data were analyzed by BIAevaluation software (GE Healthcare) to determine K_D2_. The programs used are designed to include one of the crucial effects, mass transport effect (MTL)^23^. For the analysis of binding signals of PDEγ to immobilized Tα-S*, we used Heterogeneous Ligand with MTL program with assuming that there are at least two populations of Tα-S* immobilized differently depending on which thiol site was immobilized. However, our analysis indicated that the binding of PDEγ to immobilized Tα-S* consisted of only one major component (>98%). Unfortunately, the binding signal of Tα-S* to immobilized PDEγ was not analyzed with this program, and instead, 1:1 binding with MTL program was used. However, as shown in Fig. 4b, 1:1 binding with MTL program gave a very good fit to the measured binding signals, which indicated that the binding consisted of one major component.

### Determination of K_D1_ of the PDEγ·PDEcat complex with dilution

To determine K_D1_, PDE activity was measured using purified PDE or ROS membranes at various concentrations of PDE (≤40 nM), both in the light without GTP. The activity was measured with the pH assay method using a combination glass microelectrode (MI-410, Microelectrodes, Inc.) as described previously^11,12,24^. At time 0, 5 mM cGMP was added to initiate the hydrolysis. All measurements were performed at room temperature. To measure the full PDE activity, PDEγ bound to PDEcat was digested with trypsin (final concentration, 0.1 mg/ml) for 5 min at room temperature, and the digestion was terminated by adding trypsin inhibitor at a final concentration of 0.5 mg/ml. Then, the full PDE activity measurement was initiated with adding 5 mM cGMP.

### PDE activation with Tα-S* of various concentrations

PDE activities at various concentrations of Tα-S* were measured with the pH assay method in a solution and in a ROS membrane suspension. In a solution, Tα-S* of known concentration was added to 15 nM purified PDE in K-gluc buffer in the light. At time 0, 5 mM cGMP (final concentration) was added to initiate cGMP hydrolysis. In a ROS membrane suspension, first 5 mM cGMP was added to purified ROS membranes containing 1.5, 10 or 20 μM rhodopsin in the dark, and the membranes were illuminated to activate rhodopsin fully. Then, GTPγS of known concentration was added to the membranes to initiate the cGMP hydrolysis. In the measurement in ROS membrane suspensions, concentrations of GTPγS were set so as to limit the amount of Tα-S* by the amount of GTPγS added^12^. To estimate the concentration of transducin at different concentrations of ROS membranes, we assumed that molar ratio of transducin to rhodopsin^13^ is 1/10 (for example, 2 μM transducin present in 20 μM rhodopsin membranes). In both types of preparations, solution and membrane suspension, full PDE activity was measured after trypsin digestion as described previously^12^ to determine the relative PDE activity (% max).

## Supplementary information

supplementary infomation
