# Supplementary material for: Transducin activates cGMP phosphodiesterase by trapping inhibitory γ subunit freed reversibly from the catalytic subunit in solution
Source: Sci Rep. 2019 May 10;9:7245. doi: 10.1038/s41598-019-43675-9 (PMC6510727; doi:10.1038/s41598-019-43675-9)
Supplement: Supplementary file 1 — supplementary infomation [file 41598_2019_43675_MOESM1_ESM.docx]

Supporting Information (SI)

**Transducin activates cGMP phosphodiesterase by trapping inhibitory γ subunit freed reversibly from the catalytic subunit in solution.**

Teizo Asano, Satoru Kawamura and Shuji Tachibanaki

**SI Methods**

**Formulation of dissociation of PDEγ from PDEcat.** It is expected that PDEγ is freed from PDEγ·PDEcat complex depending on its K_D_.

The reaction scheme is:

K_D1_

PDEγ + PDEcat PDEγ·PDEcat - - - (Reaction 1),

where K_D1_ is the dissociation constant of the PDEγ·PDEcat complex:

K_D1_ = [PDEγ] [PDEcat] / [PDEγ·PDEcat] - - - (s1).

Because one PDE molecule is initially composed of two catalytic subunits and two PDEγ molecules, the following relation holds:

[PDEcat]total = [PDEγ] + [PDEγ·PDEcat]

= 2 [PDE]total - - - (s2),

where [PDEcat]total is the total concentration of the catalytic subunit, and [PDE]total is the total concentration of holo-PDE. In addition, [PDEcat] should be equal to that of freed PDEγ ([PDEγ]):

[PDEcat] = [PDEγ] - - - (s3).

From Eqs. 1 – 3, we obtained a solution for [PDEcat]:

[PDEcat] = $\left( -K_{D1}+ \sqrt{K_{D1}^{2}+8K_{D1}\left[ \mathrm{PDE} \right]\mathrm{total}} \right)/ 2$ - - - (s4).

In Figs. 3, 5 and 6, we expressed each PDE activity as the relative value to the full PDE activity measured after trypsin treatment (see Methods). Relative PDE activity is obtained by dividing equation (s4) with [PDEcat]total, namely, 2[PDE]total:

Relative PDE activity = $\left( -K_{D1}+ \sqrt{K_{D1}^{2}+8K_{D1}\left[ \mathrm{PDE} \right]\mathrm{total}} \right)/ 4[PDE]total$ - - - (s5).

**Formulation of activation of PDE with Tα*** In the trapping mechanism, the reaction schemes can be written as follows.

K_D1_

PDEγ + PDEcat PDEγ·PDEcat - - - (Reaction 1),

K_D2_

PDEγ + Tα* PDEγ·Tα* - - - (Reaction 2),

where K_D1_ is the constant defined in the above, and K_D2_ is the dissociation constant of the PDEγ·Tα* complex:

K_D1_ = [PDEγ] [PDEcat] / [PDEγ·PDEcat] - - - (s1),

K_D2_ = [PDEγ] [Tα*] / [PDEγ·Tα*] - - - (s6).

Total concentrations of PDEcat ([PDEcat]total), Tα* ([Tα*]total) and PDEγ ([PDEγ]total) are expressed as follows.

[PDEcat]total = [PDEγ·PDEcat] + [PDEcat] = 2[PDE]total - - - (s7).

[Tα*]total = [PDEγ·Tα*] + [Tα*] - - - (s8).

[PDEγ]total = [PDEγ] + [PDEγ·PDEcat] + [PDEγ·Tα*]

= 2[PDE]total - - - (s9).

Using these relations, a cubic equation of [PDEcat] is obtained:

(K_D1_ - K_D2_)[PDEcat]^3^

+ (K_D1_^2^ - K_D1_ K_D2_ - 2 K_D1_ [PDEcat]total + K_D1_ [PDEγ]total - K_D1_ [Tα*]total + K_D2_[PDEcat]total

- K_D2_ [PDEγ]total)[PDEcat]^2^

- K_D1_ [PDEcat]total(2 K_D1_ - K_D2_ - [PDEcat]total + [PDEγ]total - [Tα*]total)[PDEcat]

+ (K_D1_ [PDEcat]total)^2^

= 0 - - - (s10).

The constants, K_D1_ and K_D2_, are determined experimentally (Fig. 3 and Fig. 4, respectively), and [PDEcat]total (2[PDE]total), [Tα*]total, [PDEγ]total (2[PDE]total) are all known in a measurement of PDE activity in Figs. 5 and 6. Then, equation (s10) can be solved to calculate [PDEcat] numerically at a given Tα* (actually Tα-S* in Figs. 5 and 6) concentration. PDE activity biochemically measured (Figs. 5 and 6) is expressed as the % of the full activity. To compare the biochemical and the theoretical result in Figs. 5 and 6, [PDEcat] calculated at a given [Tα*] in equation (s10) was divided by [PDEcat]total.

Estimation of PDEcat activation necessary for a single photon response. We assumed that rhodopsin (Rh) concentration in a rod is 3 mM and that there are 30,000 Rh molecules, 3,000 Transducin (Tr) molecules (Tr/Rh = 1/10) and 222 PDEcat molecules (1/2PDEcat/Rh=270) on a single surface of a disk membrane (ssDM) in mouse as an example. Based on a recent finding that 12 – 14 PDEγ·Tα* complex is necessary in ssDM to evoke a single photon response^1^, we set the Tα* concentration at 1.5 μM (corresponding to 15 Tα* in ssDM). At this concentration, we simulated the % of PDEcat activated using K_D1_ and K_D2_ obtained in membranes (K_D1_ = 55 pM and K_D2_ = 56.5 pM) in equation (1) in the main text or equation (s10) in the SI Methods. The result showed that 6.32 % of PDEcat is activate (14 PDEcat*/ssDM) and that in case no Tα* is present, i. e., in the dark, 0.16 % of PDEcat is active (0.35 PDEcat*/ssDM).

**SI reference**

1. Yue, W. W. S. et al. Elementary response triggered by transducin in retinal rods. *Proc. Natl. Acad. Sci. USA* **116**, 5144-5153 (2019)

**
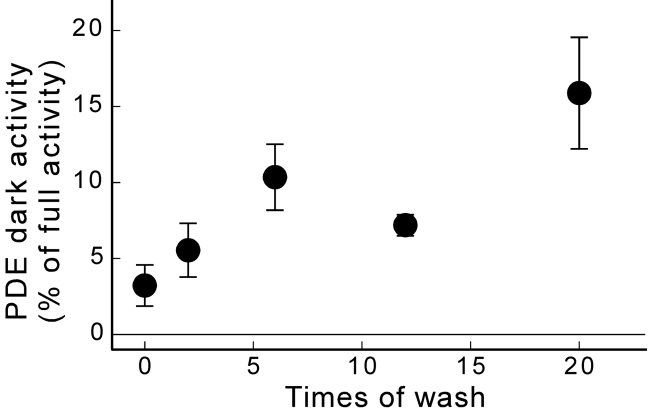
SI Figures**

Fig. S1. PDE dark activity increase with extensive washes. ROS membranes were disrupted by passing through a #27 gauge needle for 10 times and freeze-thawed. They were washed at indicated times by centrifugation (150,000 × g, 5 min) with 0.8 × K-gluc buffer, and finally resuspended in K-gluc buffer. PDE dark activity was measured with the pH assay method. The activity is expressed as the % of the full activity measured after treatment with trypsin. The results are indicated as mean ± SD (n = 5-9 except for the point at 6 washes where n = 2 and the result at this point is indicated as mean ± the range of variation).
